# Patient education, disease activity and physical function: can we be more targeted? A cross sectional study among people with rheumatoid arthritis, psoriatic arthritis and hand osteoarthritis

**DOI:** 10.1186/ar4339

**Published:** 2013-10-20

**Authors:** Răzvan G Drăgoi, Mwidimi Ndosi, Martina Sadlonova, Jackie Hill, Mona Duer, Winfried Graninger, Josef Smolen, Tanja A Stamm

**Affiliations:** 1 Department of Rheumatology, Medical University of Vienna, Clinic of Internal Medicine III, Vienna, Austria; 2 Department of Rehabilitation, Physical Medicine, Balneology and Rheumatology, “Victor Babeş” University of Medicine and Pharmacy Timişoara, Timişoara, Romania; 3 Academic & Clinical Unit for Musculoskeletal Nursing, Leeds Institute of Rheumatic and Musculoskeletal Medicine, University of Leeds, Leeds, UK; 4 Department of Internal Medicine, Division of Rheumatology and Immunology, Medical University of Graz, Graz, Austria; 5 Department of Health, University of Applied Sciences FH Campus Wien, Vienna, Austria

## Abstract

**Introduction:**

In order to target educational needs of patients more effectively, an Austrian-German educational needs assessment tool (OENAT) was developed, the educational needs of patients with rheumatoid arthritis (RA), psoriatic arthritis (PsA) and hand osteoarthritis (HOA) were described and the relationships between educational needs, gender, disease activity and function were explored.

**Methods:**

The English ENAT was adapted into Austrian-German using Beaton's cross-cultural adaptation process. Internal construct validity was assessed by Rasch analysis. Educational needs across diagnostic groups and subgroups of patients were summarized descriptively and their relationship with disease activity and physical functioning explored.

**Results:**

The sample comprised 130 RA, 125 PsA and 48 HOA patients. Their mean ages ± SD were 56 ± 14, 51 ± 11 and 64 ± 7 years for RA, PsA and HOA; disease duration was 11 ± 9, 11 ± 11 and 14 ± 9 years, respectively. More than 70% in each patient group expressed interest in receiving education about their disease.

The educational needs differed significantly between women and men in all 3 groups. In RA and PsA, female patients expressed significantly higher educational needs than men in 'movements’ and 'feelings’ domains (p=0.04 and p=0.03 for RA and p<0.01 and p=0.01 for PsA). Female patients in the HOA group had significantly higher scores on all domains except for the 'movements’. Older patients with PsA scored significantly higher than their younger counterparts in the 'pain’ domain (p=0.05). RA patients with disease duration >5 years), expressed higher educational needs in 'movements’ (p<0.01). Educational background had effects in the PsA group only, patients with basic education had greater scores than those with higher education on 'movements’ and 'arthritis process’ (p=0.01).

In the RA group, DAS28 correlated significantly with 'movements’ (r=0.24, p=0.01), 'feelings’ (r=0.22, p=0.02), and 'treatments’ (r=0.22, p=0.03). In the PsA group, all OENAT domains correlated with disease activity (DAPSA and CDAI).

**Conclusions:**

This study showed that educational needs vary with personal characteristics. Patient education may be more targeted and effective, if gender, age, educational background and disease duration are taken into account. Correlations with disease activity and function suggest that the OENAT could enable identification of 'intervention points’, which can be ideal opportunities for effective patient education.

## Introduction

Rheumatoid arthritis (RA) is a common systemic inflammatory disease characterized by the presence of destructive polyarthritis with a predisposition for affecting the small joints of the hand and feet [1]. RA leads to pain, swelling and stiffness, limitations in joint function as well as structural joint damage that impedes functioning in daily activities [2]. Psoriatic arthritis (PsA) is likewise an inflammatory joint disease that, aside from its association with psoriasis, manifests clinically in several ways, including arthritis, enthesitis, dactylitis, axial disease and skin/nail involvement [3,4]. In contrast, osteoarthritis of the hands (HOA) is regarded a non-inflammatory joint disease and constitutes one of the most prevalent musculoskeletal diseases, leading to pain in and around affected joints, as well as bony swelling, stiffness, deformity and gradual loss of function [5]. However, HOA can also occur relatively early in life, impairing the patient’s capacity to work [6]. All three above described conditions are chronic disabling diseases with impact on body functions, but also on daily activities and participation in society including productivity and employment. Although there are many definitions of patient education [7], they all indicate that it involves an interactive process between patients and professionals, aims at enabling patients’ participation in treatment and improve their coping strategies. As such, patients’ active participation in disease management and treatment of rheumatic diseases is essential and patient education is recommended as an integral part of the treatment [8].

The Austrian health care system does not offer any form of structured patient education to individuals with a rheumatic disease, and this role is left to the clinical groups and individual health professionals. As effective patient education needs to be patient-centered and tailored to individual educational needs [9], it is essential to assess the educational needs of patients before giving a specific type of education. Currently, there is no German tool for assessing educational needs of people with rheumatic diseases; furthermore the educational needs of people with rheumatic diseases in Austria have never been systematically explored. The Educational Needs Assessment Tool (the ENAT) exists in English and in six other European languages [10]. The aim of our study was to target educational needs of people with different rheumatic diseases more effectively. The specific aims were (i) to develop and validate an Austrian-German version of the ENAT (the OENAT), (ii) to use the OENAT to explore educational needs of people with RA, PsA and HOA and (iii) to search relationships between educational needs, gender, age, disease activity and functional ability.

## Methods

### Design

This study was conducted in two phases: (1) a cross-cultural adaptation and validation of the OENAT, and (2) a cross-sectional survey to explore the educational needs of people with RA, PsA or HOA, their disease activity and physical functioning. The adaptation into German followed an established process for cross-cultural adaptation of self-report measures [11], which involved (i) forward translations carried out by two bilingual translators whose mother tongue was German; (ii) synthesis of the translations, carried out by the forward translators and the recording observer producing (iii) back translations, carried out by two other bilingual translators whose mother tongue was English; (iv) the expert committee meeting to review the translations, and (v) testing the pre-final OENAT on 10 patients. Following the adaptation, 303 patients with arthritis completed the OENAT and the data were subjected to Rasch analysis to assess the construct validity and reliability of the translated tool. The validated OENAT was then used in the cross-sectional survey to assess the relationship between educational needs, disease activity and function.

### Participants

Patients with RA and HOA diagnosed according to the American College of Rheumatology (ACR) criteria [12,13], and patients with PsA diagnosed according to the criteria described by Moll and Wright, and by McGonagle *et al*. [14] were asked to participate in this study. Participation was voluntary and ethical approval was obtained from the ethical committee and internal review board of the Medical University of Vienna, Austria. Patients gave written informed consent to participate in the study and agreed that the findings of the study will be published in a scientific journal. Each patient was then asked to complete the OENAT at a routine visit to the rheumatology outpatient clinic. Patients were excluded if they (a) had any other rheumatic or neuromotor disease, (b) were unable to understand the language or study procedures or (c) were unwilling to participate.

### Assessments

The ENAT is a self-report questionnaire designed to assess the educational needs of patients with rheumatic diseases [11]. It was originally developed with patients and practitioners in the UK and comprises 39 items grouped into the following seven domains: managing pain (six items), movement (five items), feelings (four items), arthritis process (seven items), treatments (seven items), self-help measures (six items) and support systems (four items) [10]. Items consist of a statement providing a Likert scale ranging from 0 (not important at all) to 4 (extremely important). The OENAT is presented in the (see Additional file 1).

In addition to the OENAT, patients completed the German version of the Health Assessment Questionnaire (HAQ) [15,16], and the Clinical Disease Activity Index (CDAI) was calculated for the RA and PsA groups, the Simple Disease Activity Index (SDAI) for RA and the Disease Activity Index for PsA (DAPSA) [17] for the PsA group. All variables needed were recorded during the routine clinical visit at the rheumatology outpatient department.

### Statistical analyses

Rasch analysis was used to assess the internal construct validity of the OENAT and its invariance to age, gender, disease duration, educational background and diagnosis. The Rasch model specifies how data should look in order to comply with fundamental requirements of measurement, for example, unidimensionality. The observed data from the ENAT were measured against the Rasch model to assess how well they fit the model. The fit-to-Rasch model implies criterion-related construct validity, reliability and statistical sufficiency [18], thus, ordinal data from a questionnaire can be converted into interval scale and analyzed using parametric statistics [19].

Differences in educational needs were explored across gender and age groups, split at the median, and patients with different disease duration and educational backgrounds. The relationships between educational needs and disease activity and physical functioning were assessed for the RA and PSA cohorts. Mean differences (MD) and Pearson correlation (*r*_p_) with the corresponding 95% CI were calculated, where MD of 0.2, 0.5 and 0.8; and *r* of 0.1, 0.3 and 0.5 represented small, moderate and large differences in correlations, respectively [20]. All analyses were performed using SPSS software version 17 [21].

## Results

### Demographic data

In total, 303 patients participated in our study, of whom 130 had RA, 125 PsA and 48 HOA. Their demographic data were similar to the population of patients in their respective diagnostic groups [1] (Table 1). There were significant differences in mean age between the three cohorts (F(2) = 19.08, *P* <0.001): patients with HOA were older than the other two groups. There were no significant differences in disease duration (F(2) = 1.10, *P* = 0.33). The majority of patients (above 70%) in each group expressed interest in receiving education about their disease (Table 1).

**Table 1:** Demographic data of the three diagnostic cohorts

		**RA**	**PsA**	**HOA**
Female, n (%)		98 (75%)	56 (45%)	40 (83%)
Age, years, mean (SD)		56 (13.6)	51 (10.5)	64 (7)
Disease duration, years, mean (SD)		11 (9)	11 (10.9)	14 (9.1)
Educational background	Basic, n (%)	46 (35%)	32 (26%)	21 (44%)
	Secondary, n (%)	50 (39%)	66 (53%)	17 (35%)
	Above, n (%)	31 (23.8%)	23 (18.4%)	9 (18.8%)
Interest in receiving education		91 (70%)	93 (74%)	81 (89%)
How much information?	None, n (%)	6 (4.6%)	3 (2.4%)	2 (4.2% )
	Some things, n (%)	24 (18.5% )	17 (13.6% )	5 (10.4%)
	A lot of things, n (%)	24 (18.5%)	34 (27.2%)	5 (10.4% )
	Everything, n (%)	74 (57%)	71 (56.8%)	36 (75% )

### Internal construct validity and unidimensionality of OENAT

Rasch analysis of the OENAT revealed that the seven domains of the OENAT formed a unidimensional scale, suggesting the use of domain scores rather than individual item scores. In addition, the OENAT worked consistently across patients with different ages, gender, disease duration and educational backgrounds. The ordinal data from the OENAT were then converted into interval scale (for Rasch-transformed values see Additional file 2) and analyzed using parametric statistics [19].

### Educational interest and relationship to other variables

Table 2 summarizes the educational needs of people with RA, PsA and OA by gender, age, disease duration and educational background. The significant results are indicated by asterisks in the table. Gender appears to have an effect on educational needs in all the three cohorts. In the RA group, female patients expressed higher educational needs than their male counterparts in movement (*P* = 0.04) and feelings (*P* = 0.03). Like those in the RA group, female patients with PsA scored higher on movement (*P* <0.01) and feelings, (*P* <0.01). Interestingly, female patients in the HOA group had significantly higher scores on all domains except on the movement domain. Age had effects on the PsA cohort only, where older patients with PsA scored higher than their younger counterparts in the pain domain, (*P* = 0.05). Disease duration had significant effects on the RA group where patients with longer disease duration (>5 years), expressed higher educational needs in movements, (*P* <0.01). Educational background had effects in the PsA but not the other cohorts. Patients with basic education only, had greater scores than those with higher educational background especially on movements (*P* = 0.01) and arthritis process (*P* = 0.01).

**Table 2:** Summary of educational needs across diagnostic groups

**Diagnoses**	**Domains**	**Age**				**Gender**				**Disease duration**				**Educational background**			
		**Older, mean (SD)**	**Younger, mean (SD)**	**MD** **(95% CI)**	***P*-value**	**Male, mean (SD)**	**Female, mean (SD)**	**MD (95% CI)**	***P*-value**	**Longer, mean (SD)**	**Shorter, mean (SD)**	**MD (95% CI)**	***P*-value**	**Basic, mean (SD)**	**Higher, mean (SD)**	**MD (95% CI)**	***P*-value**
RA (n = 130)	Pain	15.13 (5.29)	15.42 (5.11)	-0.28 (-2.24,1.67)	0.772	14.95 (4.89)	15.4 (5.24)	-0.45 (-2.75, 1.83)	0.693	15.31 (5.10)	14.48 (5.15)	0.83 (-1.51, 3.17)	0.484	15.39 (5.17)	15.12 (5.06)	0.27 (-1.93, 2.47)	0.810
	Movement	11.50 (5.05)	11.63 (5.32)	-0.12 (-2.00, 1.75)	0.894	9.85 (5.77)*	12.12 (4.89) *	-2.27	0.040*	12.20 (4.91)*	9.02 (5.38)*	3.17 (0.99, 5.35)*	0.005*	12.05 (4.94)	10.08 (5.78)	1.97 (-0.18, 4.12)	0.073
								(-4.45, -0.10)*									
	Feelings	8.38 (5.18)	9.14 (4.27)	-0.75 (-2.44, 0.93)	0.382	7.23 (4.80)*	9.33 (4.55)*	-.2.10	0.033*	9.17 (4.72)	7.42 (4.59)	1.75 (-3.02, 2.35)	0.087	9.00 (4.60)	8.18 (4.83)	0.82 (-1.09, 2.75)	0.396
								(-4.04, -0.17)*									
	Arthritis	21.96 (5.28)	22.38 (5.38)	-0.42 (-2.36, 1.51)	0.677	21.62 (5.30)	22.44 (5.34)	-0.82 (-3.07, 1.42)	0.469	22.19 (5.54)	21.75 (4.66)	0.44 (-1.85, 2.73)	0.704	22.80 (4.84)	20.82 (5.79)	1.98 (-0.19, 4.15)	0.074
	Treatments	18.38 (6.00)	18.26 (5.89)	0.11 (-2.20, 2.41)	0.923	16.83 (5.67)	18.87 (5.94)	-2.03 (-4.66, 0.59)	0.127	18.19 (5.83)	18.53 (6.43)	-0.33 (-3.02, 2,35)	0.807	18.39 (5.41)	18.09 (7.22)	0.30 (-2.28, 2.89)	0.816
	Self-help	16.05 (5.33)	16.67 (5.06)	0.97 (-2.54, 1.30)	0.536	15.57 (4.80)	16.71 (5.24)	-1.13 (-3.33, 1.07)	0.311	16.45 (5.05)	16.11 (5.53)	0.33 (-1.87, 2.53)	0.765	16.51 (5.24)	16.20 (4.95)	1.04 (-0.57, 2.67)	0.78
	Support	7.45 (4.10)	7.73 (3.72)	-0.28 (-1.71, 1.15)	0.700	6.93 (3.77)	7.86 (3.92)	-0.93 (-2.62, 0.75)	0.276	7.94 (3.90)	6.41 (3.74)	1.52 (-0.13, 3.19)	0.072	7.82 (3.79)	6.78 (4.06)	1.04 (-0.57, 2.67)	0.20
	Total ENAT Score	96.61 (30.18)	101 (29.69)	-5.20 (-17.79,7.38)	0.412	91.18 (31.71)	102.81 (28.84)	-11.63 (-25.77, 2.51)	0.106	101.09 (29.95)	93.85 (30.00)	7.24 (-6.98,21.47)	0.314	109.92 (28.04)	96.06 (34.56)	4.86 (-9.10,18.83)	0.49
PsA (n = 125)	Pain	17.73 (4.81)*	15.50 (4.80)*	2.23 (0.03, 4.43)*	0.050*	15.20 (5.25)	16.89 (4.09)	-1.69 (-3.49, 0.10)	0.065	16.29 (4.88)	14.87 (4.64)	1.41 (-0.69, 3.51)	0.186	16.17 (0.48)	15.51 (1.31)	0.66 (-1.71, 3.04)	0.581
	Movement	10.75 (4.91)	10.39 (5.37)	0.35 (-1.93, 2.65)	0.760	9.15 (5.34)*	11.86 (4.86)*	-2.70	0.005*	10.65 (5.20)	9.65 (5.25)	1.00 (-1.17, 3.18)	0.365	11.03 (5.00)*	7.90 (5.92)*	3.12 (0.69, 5.56)*	0.012*
								(-4.59, -0.82)*									
	Feelings	9.46 (3.82)	9.17 (4.50)	0.28 (-1.58, 2.16)	0.762	7.99 (3.96)*	10.31 (4.76)*	-2.31	0.004*	9.20 (4.21)	8.90 (4.96)	0.29 (-1.53, 2.11)	0.753	9.48 (4.46)	7.74 (4.03)	1.74 (-0.31, 3.79)	0.096
								(-3.89, -0.73)*									
	Arthritis	21.40 (4.17)	20.67 (4.92)	0.72 (-1.38, 2.83)	0.491	20.22 (4.85)	21.43 (4.66)	-1.21 (-2.97, 0.53)	0.171	21.10 (4.63)	20.15 (5.04)	0.95 (-1.03, 2.94)	0.343	21.39 (4.60)	18.60 (4.40)	2.79 (0.51, 5.07)	0.017
	Treatments	19.16 (4.25)	17.40 (5.81)	1.27 (0.77, 4.28)	0.170	16.90 (5.72)	18.77 (5.29)	-1.86 (-3.95, 0.22)	0.079	17.84 (5.55)	17.56 (5.48)	0.28 (-2.07, 2.65)	0.809	18.19 (5.43)	16.12 (5.93)	2.06 (-0.63, 4.77)	0.133
	Self-help	17.17 (4.54)	16.23 (4.89)	0.94 (-1.12, 3.01)	0.373	15.84 (5.02)	17.48 (4.42)	-1.64 (-3.39, 0.10)	0.065	16.58 (4.78)	15.88 (4.75)	0.69 (-1.29, 2.68)	0.491	16.66 (4.77)	16.17 (5.21)	0.48 (-1.78, 2.76)	0.671
	Support	8.28 (3.16)	7.26 (4.28)	1.02 (-0.80, 2.84)	0.275	6.85 (4.00)	8.15 (4.10)	-1.29 (-2.80, 0.20)	0.091	7.53 (3.89)	7.20 (4.59)	0.32 (-1.39, 2.04)	0.709	7.81 (4.12)	5.97 (3.82)	1.83 (-0.11, 3.78)	0.065
	Total ENAT Score	95.67 (28.52)	105.76 (24.33)	10.09 (-4.39, 24.58)	0.179	91.23 (28.47)	104.98 (25.37)	-13.74 (-24.89, -2.60)	0.016	98.13 (26.82)	94.14 (30.22)	3.99 (-9.11,17.11)	0.546	99.54 (26.87)	85.88 (31.58)	13.66 (-1.40,28.73)	0.075
HOA (n = 48)	Pain	15.09 (5.29)	16.78 (3.50)	-1.69 (-5.72, 2.33)	0.399	8.22 (5.27)*	17.02 (3.77)*	1.77(-12.39, -5.18)*	0.001*	15.14 (5.13)	15.63 (7.26)	-0.47 (-7.01,6.06)	0.883	15.69 (5.13)	15.56 (5.30)	0.12 (-4.24, 4.49)	0.953
	Movement	4.57 (6.21)	2.85 (2.12)	1.72 (-2.00,5.44)	0.356	1.33 (0.87)	4.65 (5.75)	-3.31 (-7.47, 0.83)	0.115	3.74 (5.25)	1.76 (0.41)	1.97 (-4.25, 8.21)	0.524	4.43 (5.86)	2.31 (0.81)	2.11 (-2.11, 6.34)	0.318
	Feelings	9.12 (5.34)	9.61 (3.68)	-0.49 (-3.87, 2.88)	0.769	2.64 (4.28)*	10.62 (3.73)*	-7.98	0.001*	8.64 (5.06)	9.84 (2.84)	-1.19 (-7.24, 4.84)	0.690	9.16 (4.81)	9.59 (5.44)	-0.42 (-4.28, 3.42)	0.824
								(-10.98, -4.98)*									
	Arthritis	17.73 (7.62)	20.18 (5.40)	-2.45 (-7.51, 2.61)	0.332	9.43 (8.67)*	20.11 (5.25)*	-10.68	0.001*	17.91 (7.49)	15.16 (0.00)	2.75 (-6.14,11.65)	0.534	18.31 (6.83)	18.64 (8.41)	-0.32 (-5.97, 5.31)	0.907
								(-15.58, -5.77)*									
	Treatments	9.52 (7.63)	9.24 (8.02)	0.28 (-5.45, 6.02)	0.923	3.51 (2.68)*	10.75 (7.76)*	-7.23	0.021*	9.21 (7.80)	6.40 (0.00)	2.81 (-6.48,12.11)	0.542	9.40 (7.34)	9.71 (9.83)	-0.30 (-7.26, 6.64)	0.929
								(-13.31, -1.16)*									
	Self-help	18.98 (4.98)	20.38 (3.20)	-1.39 (-4.54, 1.74)	0.375	14.10 (7.11)*	20.43 (3.08)*	-6.32	0.001*	19.38 (5.00)	17.79 (0.00)	1.59 (-4.14, 4.45)	0.590	19.57 (4.65)	18.73 (4.11)	0.83 (-2.76, 4.44)	0.641
								(-9.58, -3.05)*									
	Support	4.83 (3.96)	5.99 (3.38)	-1.15 (-3.77, 1.45)	0.375	1.86 (2.02)*	5.80 (3.72)*	-3.93	0.006*	4.62 (3.60)	4.47 (1.96)	0.15 (-4.14, 4.45)	0.942	4.86 (3.59)	6.06 (4.57)	-1.20 (-4.05, 1.64)	0.400
								(-6.69, -1.18)*									
	Total ENAT Score	77.77 (31.65)	97.82 (21.70)	-20.04 (-48.09, 8.00)	0.155	33.94 (26.77)*	90.82 (21.82)*	-56.88	0.001*	80.02 (34.36)	71.05 (11.10)	8.96 (-32.91,50.84)	0.663	82.17 (29.18)	78.97 (41.83)	3.20 (-27.99, 4.39)	0.835
								(-79.42, -34.33)*									

MD, mean difference (older-younger for age, male-female for gender, longer-shorter for disease duration and basic-higher for disease duration). *P*-value ≤0.05 represents significance effects against the null hypothesis on 'no difference’. *Statistically significant results. Older/younger age cutoff is based on disease-specific median age; shorter/longer disease duration cutoff is based on disease-specific 25^th^ percentile. RA, rheumatoid arthritis; PsA, psoriatic arthritis; HOA, hand osteoarthritis; ENAT, Educational Needs Assessment Tool.

Cross-diagnosis comparison of overall educational needs was possible between the RA and PsA cohorts because the OENAT for two cohorts worked on a comparable scale [22]. There were no significant differences of overall educational needs between the RA and the PsA cohorts (Table 3).

**Table 3:** Comparison of educational needs between the RA and PsA cohorts

**Domain (score range)**		**Psoriatic arthritis,**	**Rheumatoid arthritis,**	**Difference**		
		**mean (SD)**	**mean (SD)**	**MD (95% CI)**	**t-test**	**p-value**
Pain	(0 to 24)	14.29 (6.69)	13.40 (6.99)	0.89 (-0.79, 2.58)	1.04	0.30
Movement	(0 to 20)	9.79 (5.67)	11.07 (5.62)	-1.28 (-2.67, 0.11)	-1.81	0.07
Feelings	(0 to 16)	8.68 (4.73)	8.50 (4.90)	0.17 (-1.01, 1.36)	0.29	0.78
Arthritis	(0 to 28)	19.44 (6.89)	20.65 (7.65)	-1.21 (-3.01, 0.59)	-1.32	0.19
Treatments	(0 to 28)	15.90 (7.59)	15.25 (8.79)	0.65 (-1.38, 2.68)	0.63	0.53
Self-help	(0 to 24)	15.76 (5.90)	13.86 (6.82)	0.91 (-0.67, 2.49)	1.14	0.26
Support	(0 to 16)	6.83 (4.40)	7.00 (4.30)	-0.17 (-1.25, 0.90)	-0.32	0.75
Total ENAT score	(0 to 164)	90.69 (32.11)	90.73 (33.6)	-0.04 (-8.18, 8.10)	-0.01	0.99

MD, mean difference (Psoriatic arthritis cohort minus rheumatoid arthritis cohort). *P*-value ≤0.05 represents significance effects against the null hypothesis on 'no difference’.

Table 4 shows the correlations between the OENAT domains and disease activity composite measures. In the RA cohort, there were small but significant correlations between the Disease Activity Score (DAS28) and the following OENAT domains: movement (*r* = 0.24, *P* = 0.01), feelings (*r* = 0.22, *P* = 0.02), and treatments (*r* = 0.22, *P* = 0.03). However, the OENAT did not correlate with the HAQ in the RA cohort. In the PsA cohort, all OENAT domains correlated with the disease activity indices for PsA (DAPSA and CDAI). In the same cohort, physical functioning (HAQ) significantly correlated with the following OENAT domains: movements (*r* = 0.38, *P* < 0.01), feelings (*r* = 0.33, *P* = 0.01), arthritis (*r* = 0.32, *P* = 0.01) and support (*r* = .28, *P* = 0.03).

**Table 4:** Correlations between educational needs, disease activity and function for RA and PsA cohorts

**Cohort**		**DAS28**		**HAQ**		**CDAI**		**SDAI**	
		***r***_**p**_	***P*-value**	***r***_**p**_	***P*-value**	**r**_**p**_	***P*-value**	***r***_**p**_	***P*-value**
RA (n = 130)	Pain	0.044	0.662	-0.039	0.689	0.030	0.689	-0.083	0.403
	Movement	0.235*	0.013*	0.175	0.056	0.139*	0.130	-0.086	0.368
	Feelings	0.216*	0.023*	0.109*	0.235	0.054	0.557	-0.005	0.954
	Arthritis	0.086	0.375	-0.044	0.635	-0.025	0.786	-0.218*	0.023*
	Treatments	0.224*	0.028*	0.014	0.891	0.082	0.409	-0.168*	0.099
	Self help	0.169*	0.087	-0.083	0.380	0.011	0.91	-0.024	0.808
	Support	0.106*	0.285	0.089	0.342	-0.037	0.697	-0.079	0.418
	Total ENAT score	0.174*	0.115	0.019	0.860	0.030	0.777	-0.130*	0.236
		DAPSA		HAQ		CDAI			
PsA (n = 125)	Pain	0.255*	0.058*	0.113*	0.391	0.260*	0.044*		
	Movement	0.470*	0.000*	0.384*	0.002*	0.509*	0.000*		
	Feelings	0.317*	0.013*	0.325*	0.009*	0.303*	0.015*		
	Arthritis	0.406*	0.002*	0.319*	0.011*	0.422*	0.001*		
	Treatments	0.330*	0.013*	0.148*	0.259	0.358*	0.005*		
	Self help	0.361*	0.004*	0.137*	0.276	0.319*	0.010*		
	Support	0.377*	0.004*	0.283*	0.026*	0.376*	0.003*		
	Total ENAT score	0.438*	0.002*	0.282*	0.043*	0.455*	0.001*		

*r*_p_ = Pearson correlation, where *r* = 0.1, 0.3 and 0.5 represent small, moderate and large effects. *P*-value ≤0.05 represents significance effects against the null hypothesis on 'no correlation’; *significant results. RA, rheumatoid arthritis; PsA, psoriatic arthritis; ENAT, Educational Needs Assessment Tool; DAS28; Disease Activity Score 28; HAQ; Health Assessment Questionnaire; CDAI; Clinical Disease Activity Index; SDAI: Simple Disease Activity Index; DAPSA, Disease Activity Index for Psoriatic Arthritis.

## Discussion

This was the first study to explore the educational needs of patients in Austria using a standard instrument (the OENAT). A high percentage of patients in each disease group expressed interest in receiving education about their arthritis. This may be due to the fact that the Austrian health care system does not routinely offer a structured form of education to patients with rheumatic diseases. Currently, education may take place informally and it very much depends on the willingness of the health professional. The use of the OENAT may facilitate the identification of patients' priority needs and enable professionals to provide relevant education to patients. The OENAT was robust at summarizing the educational needs of people with RA, PsA and HOA, and discriminated well between different patient groups. Although the RA and PsA cohorts were comparable in their mean age, disease duration and their overall educational needs, the subgroups of patients in those two cohorts had differences in specific educational needs.

The number of female patient was higher in the RA and HOA cohorts, while the PsA cohort had almost equal gender representation, which is consistent with gender distribution in the population of patients with the specified diseases [1]. A slightly larger proportion of women showed more interest in education about disease management than men. Men with arthritis have been found to prioritize work commitments over health concerns [23] and may thus have expressed less interest in some educational needs. This may lead to the conclusion that patient education should be tailored to the needs of the participants, which may also include some gender-specific aspects.

When using the ENAT in clinical practice a clinician is able to specifically target the individual needs of a patient. Female patients had significantly more interest in the movement domain (for example, in devices that would help the patient do practical things, ways to make lifting easier, ways to save energy, getting enough rest and sleep, and ways to do things that lessen wear on the joints). A referral to a relevant health professional such as an occupational therapist may be necessary. Also, the feelings domain (for example, ways to deal with stress, moods or depression) seems to be particularly important for female patients and practitioners may want to proactively assess this domain during consultations with their female patients. This survey also suggests that written patient information leaflets should also focus on specific domains such as movements and dealing with stress, moods or depression.

Age, disease duration and educational background also had small effects in subgroups of patients, affecting only one or two domains. Interestingly, RA patients with longer disease duration (>5 years) had more educational needs in the movements domain than those with a shorter duration. This may be the case for this particular patient group, but education on movement may still be as important for patients with early arthritis to go hand in hand with treatment strategies [24,25].

As expected, the educational needs in both cohorts correlated with disease activity, which indicated that patients with more active disease were more interested in education. Patients may particularly seek education in states when they are more affected by their disease. The OENAT could be used to identify time points when patients are most interested, and therefore, more receptive in receiving education; showing that this may then be an ideal opportunity for effective patient education. However, further research is needed because this study was cross-sectional and conducted at one time-point without an educational intervention. Although both the RA and PsA cohorts had comparable disease duration and mean age, it was interesting that the OENAT correlated with physical functioning in the PsA cohort only, the reason for which also needs further investigation. One reason could be that PsA can manifest itself in various different forms, such as monoarthritis, asymmetrical oligoarthritis, symmetrical or asymmetrical polyarthritis [3,26,27].

The limitation of our study is that all our data were collected from convenience samples of patients attending the clinic at one center in Austria. Cross-diagnosis comparisons of educational needs were not possible for the HOA cohort due to a small number of patients, and the fact that the OENAT for OA has slightly different properties. Another limitation is that we included only the variables of gender, age, educational background and disease duration, to characterize our patients. However, other variables could also have influenced the educational needs of patients, for example, need for support, living situation, co-morbidities et cetera. Further studies with larger sample sizes are required for more detailed subgroup analyses, for example, between women with oligoarthritis and men with polyarthritis et cetera.

## Conclusion

The OENAT has been proved to be robust in assessing the educational needs of people with RA, PsA and HOA. It is a useful instrument to guide physicians and health professionals in the development of targeted patient-centered educational programs for patients with arthritis. The assessment of the needs of individual patients and the comparison of educational needs across subgroups of patients enables clinicians and researchers to better understand patients’ needs and plan education strategies more effectively. Correlation with disease activity and function, suggest that the OENAT can enable identification of intervention points, which can be ideal opportunities for effective patient education.

Educational needs of patients with arthritis vary with personal characteristics and these should be borne in mind. They depend on factors such as gender, educational level and disease duration. By using the ENAT in other countries [10,28], these findings will enable future comparisons between these regions and different parts of the world.

## Abbreviations

CDAI: Clinical disease activity index; DAPSA: Disease activity index for psoriatic arthritis; DAS 28: Disease activity score 28; ENAT: Educational needs assessment tool; HAQ: Health assessment questionnaire; HOA: Hand osteoarthritis; MD: Mean difference; OENAT: Austrian-German educational needs assessment tool; PsA: Psoriatic arthritis; RA: Rheumatoid arthritis; SDAI: Simple disease activity index.

## Competing interests

The authors declare that they have no competing interests.

## Authors’ contributions

Conception: RGD, MN, JH, WG, JS and TAS. Data acquisition: MS, MD and TAS. Data analysis: RGD, MN, MD and TAS. Manuscript draft: RGD, MN, MD and TAS. Advice on editing of the manuscript: RGD, MN, MS, JH, MD, WG, JS, TAS. All the authors have taken an active part in the study and take responsibility for its contents, read and approved the manuscript.

## Supplementary Material

Additional file 1 The adapted Austrian-German Educational Needs Assessment Tool (OENAT) questionnaire used in the present study. Click here for file

Additional file 2 The Rasch-transformed scores used for the hand osteoarthritis (HOA) cohort in the study. Click here for file
