# Supplementary material for: Patient education, disease activity and physical function: can we be more targeted? A cross sectional study among people with rheumatoid arthritis, psoriatic arthritis and hand osteoarthritis
Source: Arthritis Res Ther. 2013 Oct 20;15(5):R156. doi: 10.1186/ar4339 (PMC3978882; doi:10.1186/ar4339)
Supplement: Additional file 1 — The adapted Austrian-German Educational Needs Assessment Tool (OENAT) questionnaire used in the present study. [file ar4339-S1.doc]

# **Austrian Educational Needs Assessment Tool**

# **Fragebogen für den Informationsbedarf bei rheumatischen Erkrankungen (OENAT)**

No.______

**Bitte beantworten Sie die folgenden Fragen:**

**Ich bin:**

| **Männlich** |  |
| --- | --- |
| **Weiblich** |  |

**Bitte geben Sie Ihr Alter IN JAHREN an: __________**

**Wie lange haben Sie Ihre rheumatische Erkrankung schon? __________**

**Wie alt waren Sie als Sie die Pflichtschule/ oder die Schule/ oder das Studium beendet haben? __________**

**Möchten Sie zum jetzigen Zeitpunkt Informationen über Dinge, die Ihnen helfen mit der rheumatischen Erkrankung umzugehen?**

| **Ja** |  |
| --- | --- |
| **Nein** |  |

| **Wenn ja, was?** |
| --- |

**Wie viel Information möchten Sie im Allgemeinen über Ihre rheumatische Erkrankung bekommen?**

| **Ich möchte gar nichts darüber wissen** |  |
| --- | --- |
| **Ich möchte etwas darüber wissen** |  |
| **Ich möchte viele Dinge darüber wissen** |  |
| **Ich möchte alles darüber wissen** |  |

**Wie viel möchten Sie jetzt über jedes der folgenden Dinge wissen? Bitte kreuzen Sie jene Spalte an, die am besten zeigt, wie Sie empfinden:**

**Dieser Abschnitt bezieht sich auf den Umgang mit Schmerz:**

| **Wie wichtig ist es jetzt für Sie mehr über die folgenden Dinge zu wissen:** | Gar nicht wichtig | Ein bißchen wichtig | Mäßig wichtig | Sehr wichtig | Äußerstwichtig |
| --- | --- | --- | --- | --- | --- |
| Die besten Medikamente für mich |  |  |  |  |  |
| Wärme oder Kälte für schmerzhafte Gelenke |  |  |  |  |  |
| Wege sich vom Schmerz abzulenken |  |  |  |  |  |
| Entspannungsmöglichkeiten |  |  |  |  |  |
| Übungen |  |  |  |  |  |
| Akupunktur, Ultraschall oder Hydrotherapie |  |  |  |  |  |

**Dieser Abschnitt bezieht sich auf Bewegung:**

| **Wie wichtig ist es jetzt für Sie mehr über die folgenden Dinge zu wissen:** | Gar nicht wichtig | Ein bißchen wichtig | Mäßig wichtig | Sehr wichtig | Äußerstwichtig |
| --- | --- | --- | --- | --- | --- |
| Hilfsmittel, die mich in praktischen Dingen unterstützen können |  |  |  |  |  |
| Möglichkeiten um das Heben von Gegenständen zu erleichtern |  |  |  |  |  |
| Möglichkeiten Kraft zu schonen |  |  |  |  |  |
| Genügend Ruhe und Schlaf bekommen |  |  |  |  |  |
| Möglichkeiten die Gelenke nicht unnötig zu belasten |  |  |  |  |  |

**Dieser Abschnitt bezieht sich auf Ihre Gefühle:**

| **Wie wichtig ist es jetzt für Sie mehr über die folgenden Dinge zu wissen:** | Gar nicht wichtig | Ein bißchen wichtig | Mäßig wichtig | Sehr wichtig | Äußerstwichtig |
| --- | --- | --- | --- | --- | --- |
| Möglichkeiten mit Stress umzugehen |  |  |  |  |  |
| Möglichkeiten mit Stimmungsschwankungen oder Depression umzugehen |  |  |  |  |  |
| Warum ich mich müde fühle |  |  |  |  |  |
| Warum ich mich niedergeschlagen oder depressiv fühle |  |  |  |  |  |

**Dieser Abschnitt bezieht sich auf Ihre rheumatische Erkrankung:**

| **Wie wichtig ist es jetzt für Sie mehr über die folgenden Dinge zu wissen:** | Gar nicht wichtig | Ein bißchen wichtig | Mäßig wichtig | Sehr wichtig | Äußerstwichtig |
| --- | --- | --- | --- | --- | --- |
| Was meine rheumatische Erkrankung verursacht haben könnte |  |  |  |  |  |
| Welche Form der rheumatischen Erkrankung ich habe |  |  |  |  |  |
| Wie die rheumatische Erkrankung meine Kinder oder Angehörige betreffen könnte |  |  |  |  |  |
| Möglichkeiten meine rheumatische Erkrankung zu behandeln |  |  |  |  |  |
| Wie meine rheumatische Erkrankung mich betrifft |  |  |  |  |  |
| Warum ich Dinge nicht mehr wie gewohnt tun kann |  |  |  |  |  |
| Was in der Zukunft passieren könnte |  |  |  |  |  |

**Dieser Abschnitt bezieht sich auf Behandlungen, die Sie von Angehörigen von Gesundheitsberufen bekommen können:**

| **Wie wichtig ist es jetzt für Sie mehr über die folgenden Dinge zu wissen:** | Gar nicht wichtig | Ein bißchen wichtig | Mäßig wichtig | Sehr wichtig | Äußerstwichtig |  | | | | |
| --- | --- | --- | --- | --- | --- | --- | --- | --- | --- | --- |
| Warum ich Medikamente nehmen sollte |  |  |  |  |  |  | | | | |
| Wie ich meine Medikamente nehmen sollte |  |  |  |  |  |  | | | | |
| Welche Nebenwirkungen meine Medikamente haben könnten |  |  |  |  |  |  | | | | |
| Warum ich Blutuntersuchungen verordnet bekomme |  |  |  |  |  |  | | | | |
| Warum ich Röntgenuntersuchungen verordnet bekomme |  |  |  |  |  |  | | | | |
| Wie mir eine Operation helfen könnte |  |  |  |  |  |  | | | | |
| Wie Hilfsmittel mit helfen könnten (Schienen, Adaptierungen, Halskrause) | | | | | | |  |  |  |  |
|  |  |  |  |  |  |  | | | | |

**Dieser Abschnitt bezieht sich auf Behandlungen, die Sie selbst durchführen können:**

| **Wie wichtig ist es jetzt für Sie mehr über die folgenden Dinge zu wissen:** | Gar nicht wichtig | Ein bißchen wichtig | Mäßig wichtig | Sehr wichtig | Äußerstwichtig |
| --- | --- | --- | --- | --- | --- |
| Alternative Behandlungen oder pflanzliche Heilmittel |  |  |  |  |  |
| Nahrungsmittel oder Vitamine, die helfen könnten |  |  |  |  |  |
| Dinge, die ich vermeiden sollte |  |  |  |  |  |
| Übungen, die ich machen sollte |  |  |  |  |  |
| Wie viele Übungen ich machen sollte |  |  |  |  |  |
| Wann ich die/den Ärztin/ Arzt, die Krankenpflegeperson oder eine/n Angehörige/n von einem anderen Gesundheitsberuf kontaktieren sollte |  |  |  |  |  |

**Dieser Abschnitt bezieht sich auf Unterstützung von anderen Personen:**

| **Wie wichtig ist es jetzt für Sie mehr über die folgenden Dinge zu wissen:** | Gar nicht wichtig | Ein bißchen wichtig | Mäßig wichtig | Sehr wichtig | Äußerstwichtig |  | | | | |
| --- | --- | --- | --- | --- | --- | --- | --- | --- | --- | --- |
| Organisationen, mit denen ich wegen meiner rheumatischen Erkrankung Kontakt aufnehmen könnte |  |  |  |  |  |  | | | | |
| Wen ich wegen finanzieller Hilfe fragen könnte |  |  |  |  |  |  | | | | |
| Wo ich Gruppen finden kann, die mit helfen, mit der rheumatischen Erkrankung umzugehen | | | | | | |  |  |  |  |
|  |  |  |  |  |  |  | | | | |
| Wie ich am meisten von einem Besuch die/den Ärztin/ Arzt, einer Krankenpflegeperson  oder eine/n Angehörige/n von einem anderen Gesundheitsberuf profitieren kann | | | | | | |  |  |  |  |
|  |  |  |  |  |  |  | | | | |

## Vielen Dank, dass Sie sich Zeit genommen haben, diesen Fragebogen auszufüllen!
