# Supplementary material for: Patient education, disease activity and physical function: can we be more targeted? A cross sectional study among people with rheumatoid arthritis, psoriatic arthritis and hand osteoarthritis
Source: Arthritis Res Ther. 2013 Oct 20;15(5):R156. doi: 10.1186/ar4339 (PMC3978882; doi:10.1186/ar4339)
Supplement: Additional file 2 — The Rasch-transformed scores used for the hand osteoarthritis (HOA) cohort in the study. [file ar4339-S2.doc]

**Austrian HOA data conversion table**

| Raw scores | Rasch Transformed Scores | | | | | | |
| --- | --- | --- | --- | --- | --- | --- | --- |
|  | Pain | Movement | Feelings | Arthritis | Treatments | Self help | Support |
| 0 | 0.00 | 0.00 | 0.00 | 0.00 | 0.00 | 0.00 | 0.00 |
| 1 | 1.59 | 0.36 | 3.08 | 0.13 | 0.71 | 0.23 | 0.99 |
| 2 | 2.73 | 0.62 | 4.72 | 0.13 | 1.28 | 0.46 | 1.67 |
| 3 | 3.48 | 0.76 | 5.74 | 0.51 | 1.56 | 0.77 | 2.20 |
| 4 | 4.09 | 0.89 | 6.56 | 0.39 | 1.99 | 0.92 | 2.58 |
| 5 | 4.62 | 0.98 | 7.18 | 0.51 | 2.27 | 1.15 | 2.96 |
| 6 | 5.15 | 1.11 | 8.00 | 0.90 | 2.42 | 1.30 | 3.26 |
| 7 | 5.60 | 1.24 | 8.41 | 1.16 | 2.56 | 1.61 | 3.56 |
| 8 | 6.13 | 1.29 | 9.03 | 1.03 | 2.98 | 1.76 | 3.87 |
| 9 | 6.66 | 1.42 | 9.64 | 1.16 | 3.13 | 2.07 | 4.25 |
| 10 | 7.19 | 1.47 | 10.05 | 1.28 | 3.27 | 2.30 | 4.63 |
| 11 | 7.80 | 1.60 | 10.67 | 1.41 | 3.41 | 2.68 | 5.08 |
| 12 | 8.48 | 1.69 | 11.49 | 1.67 | 3.70 | 3.30 | 5.61 |
| 13 | 9.24 | 1.78 | 12.31 | 1.80 | 3.84 | 7.82 | 6.45 |
| 14 | 10.07 | 1.91 | 12.92 | 2.06 | 4.12 | 10.20 | 7.96 |
| 15 | 10.98 | 2.09 | 14.15 | 2.31 | 4.26 | 12.58 | 10.77 |
| 16 | 11.89 | 2.31 | 16.00 | 2.44 | 4.55 | 14.80 | 16.00 |
| 17 | 12.87 | 2.71 |  | 4.62 | 4.83 | 16.56 |  |
| 18 | 13.93 | 3.64 |  | 8.73 | 5.12 | 17.79 |  |
| 19 | 14.99 | 7.07 |  | 11.17 | 5.40 | 18.79 |  |
| 20 | 16.13 | 20.00 |  | 10.79 | 5.83 | 19.71 |  |
| 21 | 17.41 |  |  | 15.16 | 6.40 | 20.47 |  |
| 22 | 18.93 |  |  | 16.70 | 7.53 | 21.47 |  |
| 23 | 21.05 |  |  | 18.11 | 9.10 | 22.62 |  |
| 24 | 24.00 |  |  | 19.65 | 11.09 | 24.00 |  |
| 25 |  |  |  | 21.06 | 13.36 |  |  |
| 26 |  |  |  | 22.86 | 15.63 |  |  |
| 27 |  |  |  | 25.05 | 19.90 |  |  |
| 28 |  |  |  | 28.00 | 28.00 |  |  |

For the RA and PsA sets we used the Rasch transformed scores published by: Ndosi M, Tennant A, Bergsten U et al. Cross-cultural validation of the Educational Needs Assessment Tool in RA in 7 European countries. *BMC Musculoskelet Disord* 2011;12:110.
